# Increased Expression of PDK4 Was Displayed in Gastric Cancer and Exhibited an Association With Glucose Metabolism

**DOI:** 10.3389/fgene.2021.689585

**Published:** 2021-06-17

**Authors:** Bin Liu, Yang Zhang, Jian Suo

**Affiliations:** Department of Gastrocolorectal Surgery, The First Hospital of Jilin University, Changchun, China

**Keywords:** PDK4, gastric cancer, glucose metabolism, tumor-infiltrating immune cells, prognosis, biomarker

## Abstract

Previous studies reported that pyruvate dehydrogenase kinase 4 (PDK4) is closely related to diabetes, heart disease, and carcinomas. Nevertheless, the role of PDK4 in gastric cancer (GC) occurrence and development is yet poorly understood. Our experiments were taken to evaluate PDK4’s function in GC. The Cancer Genome Atlas tumor genome map database was employed to validate the levels of PDK family in different grades and stages of GC. The survival ratio of PDK families in GC was detected by the Kaplan–Meier plotter database. The links existing in the expression of PDK family and the level of tumor-infiltrating immune cells were investigated by tumor immunity assessment resource (TIMER). PDK4-associated signal pathways in GC were analyzed by the Kyoto Encyclopedia of Genes and Genomes pathway analysis. PDK4 mRNA level in the GC cells was measured by qRT-PCR. Cell counting kit-8 and Transwell assays were separately carried out to evaluate PDK4-induced influence on GC cell proliferation, migration, and invasion. Our data suggested that GC cells highly expressed PDK4, and PDK4 expression presented a significant relation with the staging, grade, and survival rate of GC. PDK4 expression presented a positive correlation with the types of different infiltrating immune cells, comprising B cells, CD4^+^ T cells, and dendritic cells. Meanwhile, PDK4 expression exhibited a strong association with macrophages. Survival analysis revealed that the expression of PDK4 displayed a relationship with the prognosis of patients. Therefore, PDK4 was liable to be a biomarker for prognosis. Our results further displayed that PDK4 might modulate the glycolysis level in GC cells, and its expression was associated with GC cell proliferation, migration, and invasion. These data may provide insights into designing a new treatment strategy for GC.

## Introduction

Gastric cancer (GC) deriving from the gastric mucosa is one of the deadliest occurring malignancies which threaten patients’ life worldwide (Gu et al., 2020a). As the third largest inducer of global carcinoma deaths, GC has brought a huge burden on public health (Graham, 2015; Ishaq and Nunn, 2015). The metastasis of GC is the main reason affecting the patients (Van Cutsem et al., 2016), and the development of carcinoma is closely related to the energy supply. Tumor cells primarily acquired energy by glycolysis, which resulted in a great quantity of lactic acid and a small amount ATP, distinguishing from oxidative phosphorylation occurring in the mitochondria of normal cells (Zheng, 2012). Later, the phenomenon of neoplasm uptake energy was defined as Warburg effect, usually arising in numerous tissues of the neoplasm and promoting tumor cell proliferation, invasion, and metastasis (Pelicano et al., 2006; Chen et al., 2007). Several reports have displayed that the Warburg effect, along with its dependence on tumor cells, relied on the intracellular and extracellular environment (Spencer and Stanton, 2019; Yang and Li, 2019). The levels of lactic acid, glycolytic enzymes, and hypoxia-inducible factor-1 are closely related to tumor proliferation and metastasis (Zhong et al., 1999; Lincet and Icard, 2015). What is more, glycolysis could give rise to lactic acid, and the accumulation of lactic acid would generate an acidic microenvironment instead. Under acidic conditions, the extracellular matrix was extremely unstable, and thus this boosted cancer cell metastasis (Dhup et al., 2012). Therefore, targeting the metabolism of cancer cells was a feasible root to ameliorate and unearth new anti-cancer strategies.

The change in aerobic glycolysis is a recognized feature of energy metabolism in cancer cells and is called the Warburg effect. Increased glycolysis is the main energy source for cancer cells to use this metabolic pathway to produce ATP. The glycolytic pathway is regarded as the target of cancer treatment. Cancer cells maintain a high rate of glycolysis. Pyruvate dehydrogenase kinase (PDK) contributes to this phenomenon, which is conducive to apoptosis resistance and cell transformation. Pyruvate dehydrogenase (PDH) was the key executor in facilitating pyruvate entering the tricarboxylic acid cycle. The activity of PDH could be inhibited by PDK, which was responsible for the conversion from mitochondrial oxidation to cytoplasmic glycolysis (Sutendra and Michelakis, 2013). Dichloroacetate (DCA) was an inhibitor of PDK and could alter the metabolism in the opposite direction (Michelakis et al., 2008). DCA could induce apoptosis but hinder tumor growth and decrease the level of HIF1A controlling the response of hypoxia (Sutendra et al., 2013). PDK1, PDK2, PDK3, and PDK4 are four human kinases of the PDK family (Cesi et al., 2017). PDK4 was reported to display an inhibition of pyruvate oxidation and intervene the change from glucose metabolism to fatty acid metabolism (Liu et al., 2017; Pettersen et al., 2019). Previously, some reports revealed that PDK4 have shown a close association with diabetes, heart disease, and carcinomas (Holness et al., 2002; Furuyama et al., 2003). Moreover, PDK4 was also decreased in multiple carcinomas as previously described, such as prostate carcinoma, breast carcinoma, lung carcinoma, and liver carcinoma (Grassian et al., 2011; Mengual et al., 2014; Sun et al., 2014; Choiniere et al., 2017). Besides this, PDK4 could contribute to the inhibition of cell proliferation and induction of apoptosis in lung and breast carcinoma (Grassian et al., 2011; Li et al., 2017). Additionally, the absence of PDK4 could motivate the EMT program and facilitate ovarian carcinoma cell migration and invasion (Sun S. et al., 2017). Nevertheless, the function of PDK4 in GC was yet elusive.

Here the correlation between PDK family and clinicopathological characteristics and prognosis was analyzed by sequencing data sets. The Kyoto Encyclopedia of Genes and Genomes (KEGG) and The Cancer Genome Atlas (TCGA) data revealed the possible molecular functions of PDK4. Besides that, the association existing in PDK4 expression and tumor-infiltrating immune cells was evaluated. We decreased PDK4 expression in GC cells and then validated the impacts on glycolysis. Moreover, our data revealed that an increase in the expression of PDK4 was displayed in GC, and a high level of PDK4 would lead to a lower rate of overall survival (OS) and a higher rate of recurrence. The inhibited PDK4 greatly reduced GC cell proliferation, migration, and invasion.

## Materials and Methods

### Public Database

We collected mRNA expression profiles from TCGA GC cohort^1^ and downloaded clinical information from the TCGA data portal (Gu et al., 2021b). The expression level of the PDK family in GC was determined by utilizing the tumor immunity assessment resource (TIMER)^2^ and the TCGA databases.

### Analysis of PDK Family and Clinicopathological Characteristic

The clinicopathological data of GC patients was downloaded from TCGA. We selected GC tissue samples with clinical pathological data, including classification, staging, and depth of invasion, for further analysis. The association existing in PDK family expression levels and clinicopathological value was analyzed by chi-square test.

### Analysis of OS

The Gene Expression Profile Interactive Analysis (GEPIA)^3^ was employed to analyze the data from TCGA database and the expression of genotype in tissues (Gu et al., 2020b). Kaplan–Meier plotter online database^4^ could validate the impact of each gene on the survival rate of GC patients. GEPIA and Kaplan–Meier plotter online databases were both taken to evaluate the prognostic value of PDK4 in patients with GC. The inspection probe number used by PKD4 was 205960_at. The *P*-value of log-rank and hazard ratio with 95% confidence intervals were measured.

### GO Annotation and KEGG Pathway Enrichment Analyses

For exploring PDK4’s function, we employed Enrichr database^5^ for Gene Ontology (GO) annotation and KEGG pathway enrichment analysis. Significant statistical difference is indicated as *P* < 0.05 (Zhang et al., 2020; Gu et al., 2021a).

### Cell Culture

Normal human gastric epithelium cell line (GES-1) and human GC cells (SGC-7901, MGC−803, HGC 27, and AGS) were acquired from the Cell Bank of Institute of Biochemistry and Cell Biology at the Chinese Academy of Sciences (Shanghai, China). They were maintained in Dulbecco’s modified Eagle’s containing 10% fetal bovine serum (FBS) and 1% P/S (Gibco, United States) under 37°C humidified incubator with 5% CO_2_.

### RNA Extraction and Quantitative Reverse Transcription PCR

Overall, the RNA of cells was isolated by TRIzol. The extracted RNA was reversely transcribed into cDNA by cDNA synthesis kit (TaKaRa, Japan). Primers designed by Primer 5.0 software were synthesized by Invitrogen. PCR was carried out by utilizing SYBR GREEN on an ABI 7300 plus real-time system. The 2^–ΔΔ*Ct*^ method was taken to normalize the PDK4 mRNA expression levels in GC cells and control. The RNA primers were as follows: PDK4, 5′-GGAGCATTTCTCGCGCTACA-3′ (forward), 5′-ACAGGCAATTCTTGTCGCAAA-3′ (reverse); GAPDH, 5′-CTGGGCTACACTGAGCACC-3′ (forward), 5′-AAGTGGTCGTTGAGGGCAATG-3′ (reverse). GAPDH was used as a reference.

### Transient Transfection

SiRNA (si-PDK4) (100 nM, GenePharma, China) was introduced into AGS cells to silence PDK4, and si-NC was selected as the negative control (NC) for si-PDK4.

Lipofectamine 2000 kit (Invitrogen, Carlsbad, United States) was used for cell transfer. At 48 h after transfection, different transfected cells were obtained for the next experiment. The sequence of the siRNA is as follows: si-PDK41: CGCCAACATTCTGAAGGAAATTGAT; si-NC: UUCUCCGAACGUGUCACGUTT.

### Cell Proliferation Assay

CCK-8 kit (Dojindo, Japan) was employed to determine the ability of cell proliferation in GC cells. The indicated number of GC cells was plated in a 96-well plate and then subjected to different treatments at the indicated time. The OD value of 450 nm was detected after incubation with CCK-8 solution on Fluoroskan Ascent Fluorometer (Thermo Fisher Scientific, Finland).

### Transwell Assays

Cell migration and invasion were measured using transwell chamber (8-μm pore size; Corning Co., Corning, United States) without/with Matrigel (Becton Dickinson, New York, United States). At 48 h after transfection, cells in 200 μl of medium, in the absence of serum, were added into the upper chambers. Then, 600 μl of medium containing 10% FBS was added into the lower chambers. At 24 h post-incubation, a cotton-tipped swab was applied to remove the cells staying in the lower chamber. All the cells removed were fixed by methanol for 10 min and stained with DAPI for 30 min at room temperature and protected from light. The number of cells was calculated under an inverted phase-contrast microscope (Olympus, Tokyo, Japan) (Kunig et al., 2020).

### Statistical Analysis

All derived data were shown as the mean ± SEM from three separate experiments in triplicate. GraphPad Prism 5 software (GraphPad Software, Inc.) was carried out to perform data analysis. Student’s *t*-test was conducted to perform comparisons between the two groups. A comparison amid three groups was evaluated by one-way ANOVA (Shi et al., 2020), subsequently subjecting to Kaplan–Meier method with log-rank test to test the survival curves (Sun Y. et al., 2017; Gu et al., 2018). Significant statistical difference is indicated as *P* < 0.05 (Chen et al., 2020, 2021).

## Results

### PDK Family and Clinicopathological Parameters

**FIGURE 1:**
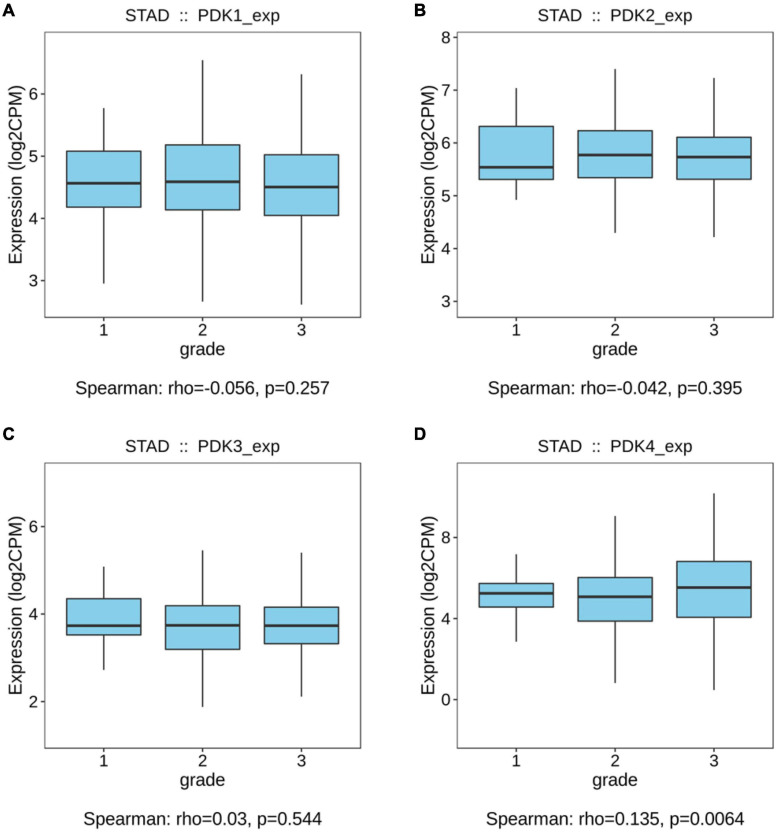
PDK4 expression exhibited a significant correlation with neoplasm grade of STAD. **(A–D)** Analysis of the relationship between tumor grade and PDK1, PDK2, PDK3, and PDK4 expression in STAD patients on the basis of The Cancer Genome Atlas database.

**FIGURE 2:**
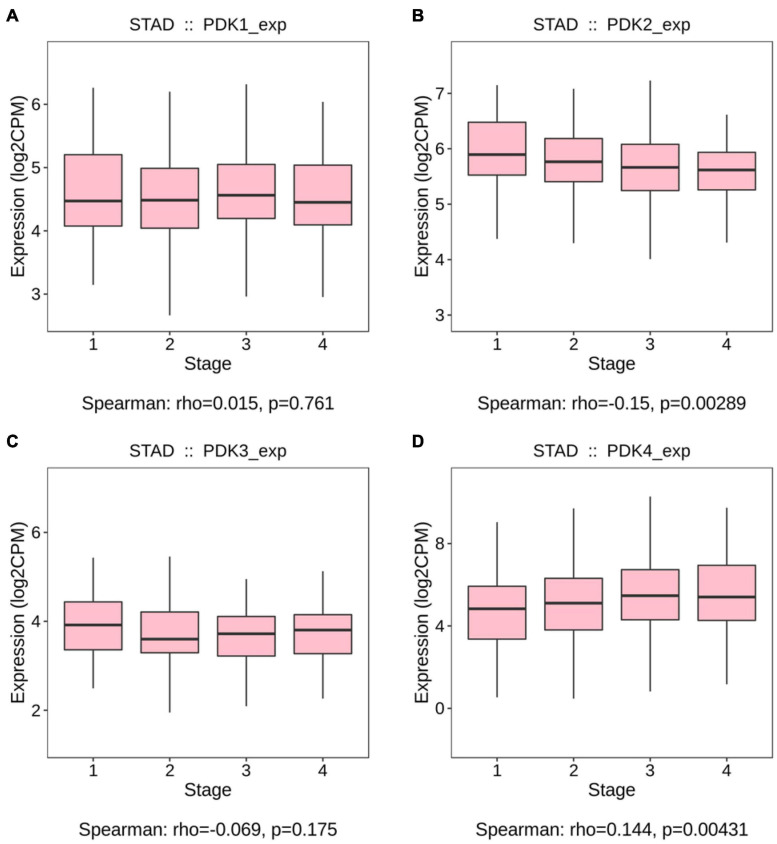
PDK2 and PDK4 expression was greatly associated with the tumor stage of STAD. **(A–D)** The Cancer Genome Atlas cohort was employed to evaluate the links between PDK1, PDK2, PDK3, and PDK4 expression and the tumor stage of STAD.

The links between the expression of PDK family and clinicopathological parameters, including the grade and the stage of tumor, were analyzed on the basis of the clinical and pathological data of STAD patients. No significant statistical relationship existed between PDK1, PDK2, and PDK3 expression and tumor grade (*P* = 0.257, *P* = 0.395, *P* = 0.544, Figures 1A–C). Importantly, PDK4 expression exhibited a large association with the grade of tumor (*P* = 0.0064, Figure 1D). In grade 3, the expression level of PDK4 was the highest. Similarly, no significant relationship was shown between PDK1 and PDK3 expression and tumor stage (*P* = 0.761, *P* = 0.175, Figures 2A,C). PDK2 and PDK4 expression had shown a great correlation with tumor stage (*P* = 0.00289, *P* = 0.00431, Figures 2B,D). Interestingly, the expression of PDK2 was highest in stage 1, while the expression of PDK4 was lowest in stage 1.

### Identification of Prognosis of PDK Family

**FIGURE 3:**
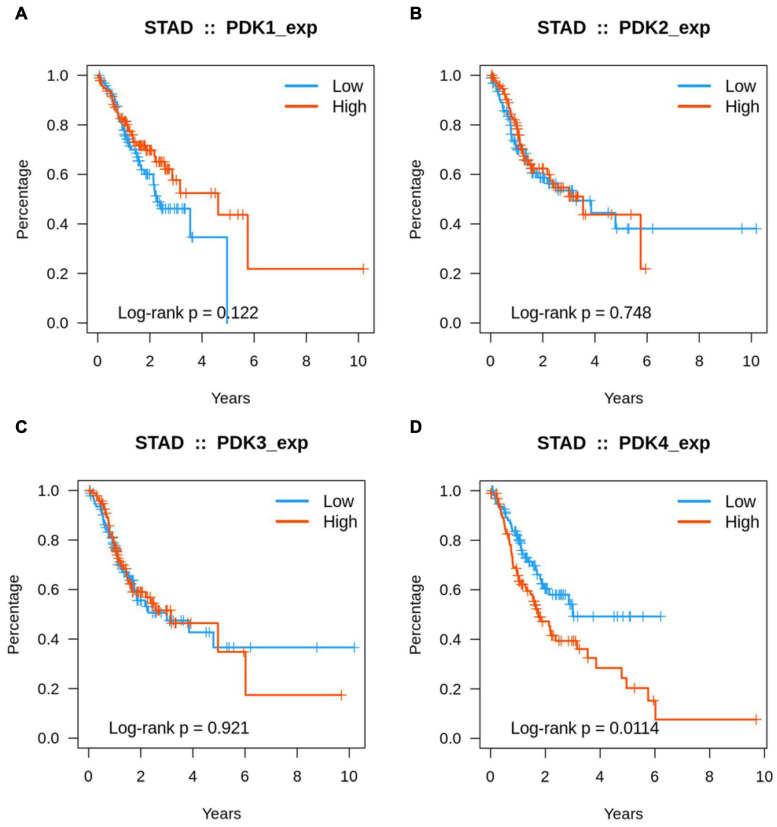
A high expression of PDK4 indicated a lower survival rate. **(A–D)** Kaplan–Meier analysis of the association between PDK1, PDK2, PDK3, and PDK4 expression and the survival rate of STAD patients.

Kaplan–Meier analysis was used to identify the prognosis parameter of PDK1, PDK2, PDK3, and PDK4. Our data revealed that PDK1, PDK2, and PDK3 expression had no significant relationship with the STAD patients’ survival rate (Figures 3A–C). Interestingly, the PDK4 expression was evidently related with survival rate (Figure 3D). Further data revealed that a lower survival rate was demonstrated in STAD patients with a high expression of PDK4. According to the survival analysis, the higher the expression of PDK4, the higher the probability of a poorly prognostic status.

### PDK4 Significantly Correlated With Tumor-Infiltrating Immune Cells in GC

**FIGURE 4:**
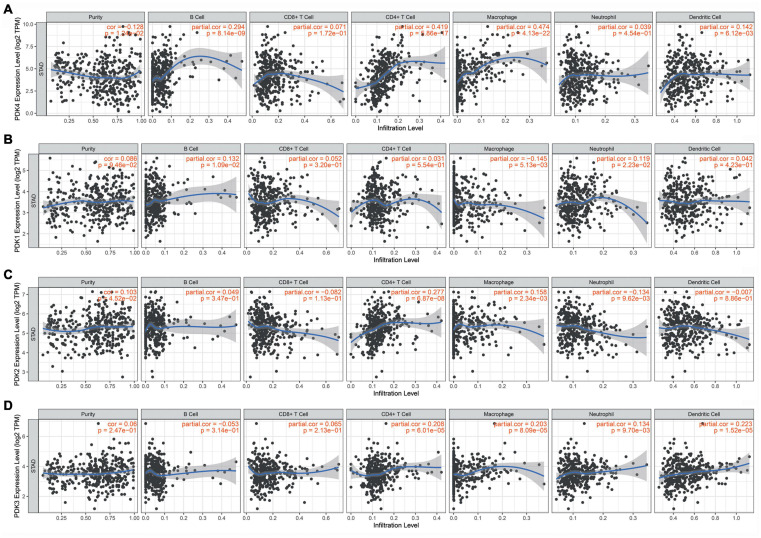
PDK4 was significantly associated with tumor-infiltrating immune cells in gastric cancer (GC). Obvious association of the PDK family with the level of tumor immune filtration in GC. PDK4 **(A)**, PDK1 **(B)**, PDK2 **(C)**, and PDK3 **(D)**.

The hidden links between PDK1, PDK2, PDK3, and PDK4 expression and tumor-infiltrating immune cells in GC were assessed by the TIMER database. The data suggested that PDK4 expression had positive links with different infiltrating immune cells level, comprising B cells (*r* = 0.294, *P* = 8.14e–09), CD4^+^ T cells (*r* = 0.419, *P* = 5.86e–17), and dendritic cells (*r* = 0.142, *P* = 6.12e–03) and exhibited a strong correlation with macrophages (*r* = 0.474, *P* = 4.13e–22) (Figure 4A). At the same time, our results showed that PDK1 (Figure 4B), PDK2 (Figure 4C), and PDK3 (Figure 4D) were not significantly related to immune cells. Collectively, our data suggested that PDK4, along with the co-expressed genes, probably participated in immune response in the microenvironment of tumor *via* exerting an effect on immune cells, especially macrophages.

### PDK4 Was an Adverse Factor for GC Prognosis

**FIGURE 5:**
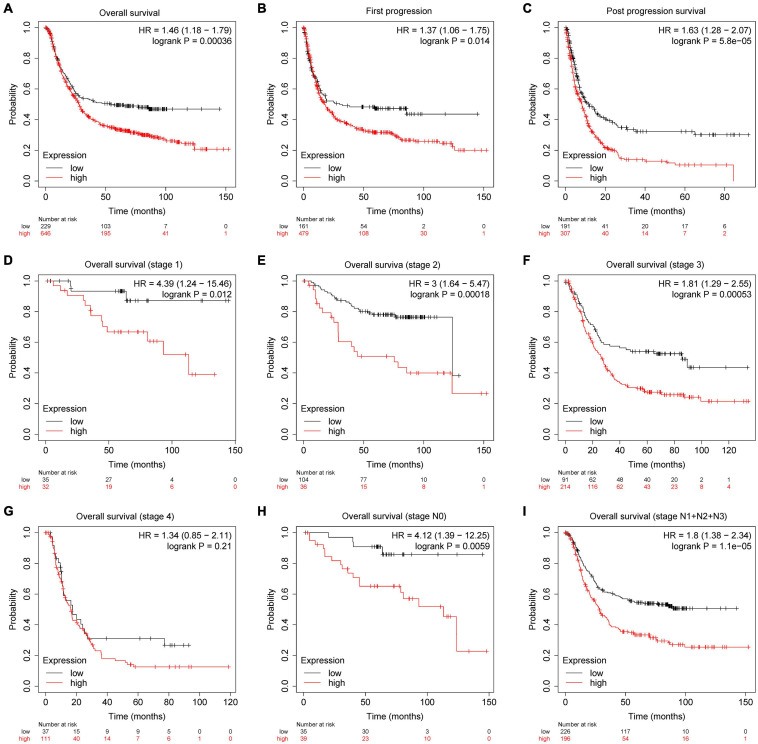
PDK4 was significantly related to the survival of gastric cancer (GC) patients. Kaplan–Meier plotter was used to analyze the relationship between the expression level of PDK4 and the overall survival. **(A)** First progression, **(B)** post-progression survival, **(C)** post-progression survival, **(D)** overall survival (stage 1), **(E)** overall survival (stage 2), **(F)** overall survival (stage 3), **(G)** overall survival (stage 4), **(H)** overall survival (stage N0), and **(I)** overall survival (stages N1 + N2 + N3) of gastric cancer patients.

The prognostic parameter of PDK4 in GC was analyzed by utilizing GEPIA data and the Kaplan–Meier plotter database accompanied by GEO data. The results showed that, compared with gastric cancer patients with low PDK4 expression, the OS of gastric cancer patients with high PDK4 expression had a significantly lower first progression survival and post-progression survival (*P* = 0.00036, *P* = 0.014, *P* = 5.8e-05 (Figures 5A–C). Compared with patients with gastric cancer and low PDK4 expression, patients with stage 1, 2, and 3 gastric cancer with high PDK4 expression had a significantly lower OS (*P* = 0.012, *P* = 0.0018, *P* = 0.00053, *P* = 0.21; Figures 5D–G). Compared with patients with gastric cancer with low PDK4 expression, patients with stage N0 gastric cancer with high PDK4 expression had a significantly lower OS (Figure 5H, *P* = 0.0059). Compared with patients with gastric cancer with low PDK4 expression, the OS of N1 + N2 + N3 gastric cancer patients with high PDK4 expression was significantly lower (*P* = 1.1e-05, Figure 5I). In summary, we infer that the high expression of PDK4 is related to the poor prognosis of gastric cancer.

### Functional Analysis of PDK4

**FIGURE 6:**
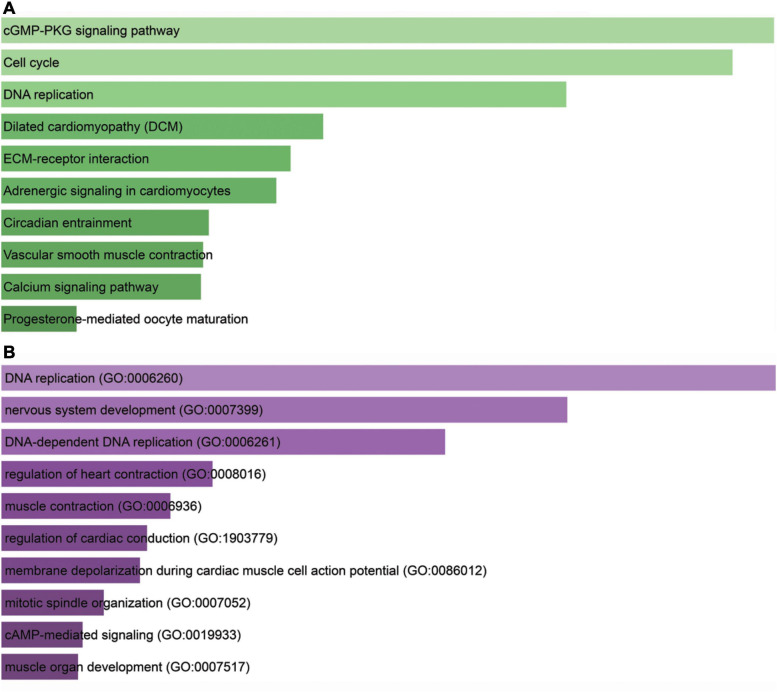
Bioinformatics analysis for PDK4. **(A)** Kyoto Encyclopedia of Genes and Genomes analysis of enriched pathways of PDK4. **(B)** Gene Ontology analysis of PDK4. The X-axis indicated gene count; the Y-axis meant enriched pathway or GO term. The color represents the *P*-value.

KEGG analysis showed that cGMP-PKG signaling pathway, cell cycle, DNA replication, dilated cardiomyopathy, extracellular membrane–receptor interaction, adrenergic signaling in cardiomyocytes, circadian entrainment, vascular smooth muscle contraction, calcium signaling pathway, and progesterone-mediated oocyte maturation were the most important pathways (Figure 6A). GO analysis data revealed that PDK4 primarily took part in the positive modulation of DNA replication (GO:0006260), nervous system development (GO:0007399), DNA-dependent DNA replication (GO:0006261), regulation of heart contraction (GO:0008016), muscle contraction (GO:0006936), regulation of cardiac conduction (G0:1903779), membrane depolarization during cardiac muscle cell action potential (GO:0086012), mitotic spindle organization (GO:0007052), cAMP-mediated signaling (GO:0019933), and muscle organ development (GO:0007517) (Figure 6B).

### Highly Expressed PDK4 mRNA Was Observed in GC Cell

**FIGURE 7:**
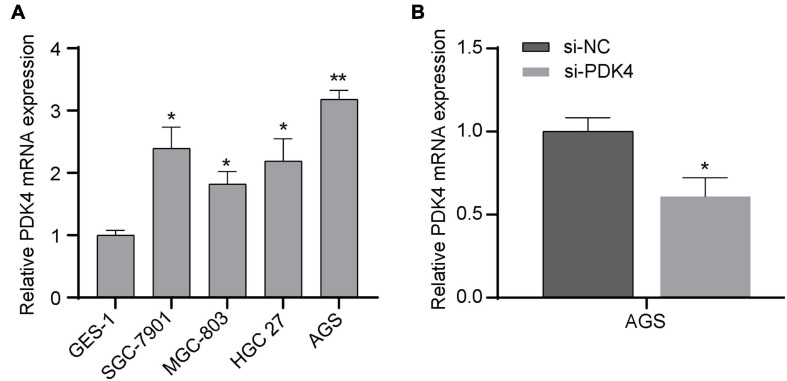
Upregulated PDK4 was shown in gastric cancer (GC) cells, and the si-PDK4 knockdown efficiency was high. **(A)** qRT-PCR analysis of PDK4 expression in four GC cell lines and normal cell lines. **(B)** qRT-PCR analysis of PDK4 expression in si-PDK4-transfected AGS cells. *P < 0.05, **P < 0.01.

In order to verify whether the expression of PDK4 in GC cell lines was also upregulated, we also conducted qRT-PCR to measure and compare the PDK4 level in several GC cell lines and human normal gastric cell lines. Our data suggested that PDK4 was significantly upregulated in the following GC cell lines compared to normal cell lines: SGC-7901, MGC-823, HGC 27, and AGS (*P* < 0.05, Figure 7A). Our data was in line with the results of the database, that is, the expression of PDK4 in GC was upregulated. In addition, we found that relative to the si-NC-transfected group, the PDK4 expression level in the si-PDK4-transfected AGS cells was reduced (*P* < 0.05, Figure 7B).

### PDK4 Expression Exerted an Effect on GC Cell Proliferation, Invasion, and Migration

**FIGURE 8:**
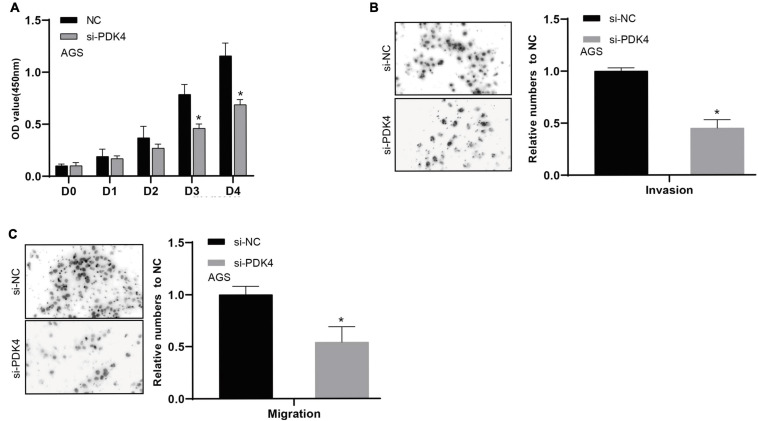
siRNA-mediated silencing of PDK4 expression inhibits the proliferation, invasion, and migration of STAD cells. **(A–C)** Assessment of the capability of cell proliferation, cell invasion, and cell migration in si-PDK4-transfected AGS cells. *P < 0.05.

The CCK-8 analysis data revealed that silencing the PDK4 expression contributed to inhibited GC cell proliferation. Relative to the si-NC-treated group, the si-PDK4-transfected AGS cells showed reduced proliferation ability (*P* < 0.05, Figure 8A). In order to further study the correlation between the low PDK4 expression and the invasion and migration of GC cells, we conducted Transwell assay. Our data suggested that the cell number of invasion (*P* < 0.05, Figure 8B) and migration (*P* < 0.05, Figure 8C) in the si-PDK4-transfected AGS cells was fewer than that in the si-NC-transfected group.

## Discussion

Currently, GC has become a global issue concerning human health. The study of GC never stops. PDK4 was reported to act as an essential mediator of cell metabolism and to display importance in the development of tumorigenesis and metastasis. Many potential targets in cancers were developed and studied (Gu and Chen, 2020; Bao et al., 2021). Previous reports had revealed that upregulating PDK4 could affect numerous carcinomas, such as lung carcinoma, breast carcinoma, ovarian carcinoma, and colon carcinoma (Yang et al., 2019). Overexpression of PDK4 also brought about drug resistance, survival, and metastasis (Wang et al., 2019). A new study suggested that PDK4 plays an oncogene role in GC, consistent with our research. Here our results implied that the highly expressed PDK4 mRNA was observed in the cell lines of GC, suggesting that PDK4 might promote GC proliferation. In addition, we validated the association between the expression of PDK4 and clinicopathological characteristics, including the prognosis and survival rate of patients, by analyzing the clinical samples of GC. The data indicated that PDK4 might be regarded as a newly generated indicator for GC patients’ prognosis. TCGA data-based pathway enrichment and gene correlation analysis revealed that GC cells might enrich PDK4 *via* glycolysis-related pathways.

It is known that changes in energy metabolism functions importantly in carcinoma progression. The capability of accelerating the uptake and the oxidation of glucose is characteristic of most malignant neoplasms. Most cancer cells prefer glycolysis rather than mitochondrial oxidative phosphorylation (OXPHOS). Glycolysis is the primary energy supply pathway for the rapid proliferation of tumor cells, allowing them to adapt to the hypoxic environment and further raise their malignant potential (Chen et al., 2007; Zheng, 2012). In humans, PDK has four isoforms (1–4). According to reports, SPRY4-IT1 promotes the survival of colorectal cancer cells by regulating PDK1-mediated glycolysis. PDK2-enhanced glycolysis promotes fibroblast proliferation in thyroid-associated ophthalmopathy. In the study of chemoresistance in gastric cancer, PDK3 is highly expressed to promote glycolysis in chemoresistant cancer cells. PDK4 was shown to facilitate the glycolysis of aerobic and cell proliferation of neoplasms (Yu et al., 2017). Studies have shown that m^6^A regulates the glycolysis of cancer cells through PDK4 (Li et al., 2020). We assumed that it may result from the fact that PDK4 reduced the content of acetyl CoA, a precursor of the synthesis of fatty acid and the production of energy. Nevertheless, tumor cells were engaged in a sole metabolic reprogramming, which was identified as the Warburg effect (Chen et al., 2007). Thus, the content of acetyl CoA was not essential. Therefore, PDK4 acts as a balancer between glycolysis and oxidative phosphorylation. Nevertheless, the particular mechanism relating to PDK4 in carcinoma still needed to be explored. PDK4 acts as an important gene relating to glucose metabolism and participates in the control of glucose metabolism and mitochondrial respiration (Guda et al., 2018). Therefore, we want to know whether PDK4 affects the proliferation and invasion of GC *via* modulating glycolysis. Our research shows that, on the basis of TCGA data for pathway enrichment and gene correlation analysis, PDK4 is usually reduced in the clinical samples of GC. GC may enrich PDK4 through glycolysis-related pathways, thereby affecting the progression of GC. Additionally, the function of PDK4, which was involved in glycolysis, was identified through gene set enrichment analysis.

Most studies focused on PDK1, PDK2, or PDK3. Zhang et al. (2006) found that the most frequently occurring isoforms of PDK were PDK2 and PDK4. However, PDK4 was not widely validated in its transformation. For exploring the functions and prognostic implication of PDK4 in GC, we conducted conclusive experiments. To evaluate the role of PDK4 in tumorigenesis, we ablated PDK4 expression by siRNAs in human GC cells. Our data indicated that a high expression of PDK4 brought about a short OS rate of patients with GC. Knockdown of PDK4 attenuated the ability of GC cell proliferation, invasion, and metastasis *in vitro*. Additionally, due to the increased expression in gastric cancer, PDK4 was a probable target for the treatment of GC.

This study has some limitations. First, it is necessary to verify the regulation of PDK4 glycolysis in GC cells. Second, the protein expression level of PDK4 needs to be tested internally in GC clinical samples. In future studies, we will collect more GC clinical samples to detect the protein expression level of PDK4 and its prognostic value. We also plan to further explore the *in vivo* function of PDK4 in animal models.

Taken together, not only is PDK4 associated with the glycolysis and proliferation of neoplasm but also it exerted an effect on the development and prognosis of neoplasm. Our data displayed that overexpressing PDK4 in GC exhibited an association with clinicopathological parameters and poor prognostic status. Highly expressed PDK4 presented an intimate relation with the level of infiltrating immune cells. Our findings demonstrated that PDK4 was a biomarker for GC prognosis and a promising target in the therapy of GC.

## Data Availability Statement

The original contributions presented in the study are included in the article/supplementary material, further inquiries can be directed to the corresponding author/s.

## Author Contributions

JS designed the study. All authors collected the data, performed the experiments, wrote the manuscript, and approved the final version of the manuscript.

## Conflict of Interest

The authors declare that the research was conducted in the absence of any commercial or financial relationships that could be construed as a potential conflict of interest.

## Footnotes

1 http://cancergenome.nih.gov/

2 https://cistrome.shinyapps.io/timer/

3 http://gepia.cancer-pku.cn/

4 http://kmplot.com/analysis/

5 https://maayanlab.cloud/Enrichr/
